# EGF-reduced *Wnt5a* transcription induces epithelial-mesenchymal transition via Arf6-ERK signaling in gastric cancer cells

**DOI:** 10.18632/oncotarget.3133

**Published:** 2015-03-12

**Authors:** Yujie Zhang, Jun Du, Jianchao Zheng, Jiaojing Liu, Rui Xu, Tian Shen, Yichao Zhu, Jun Chang, Hong Wang, Zhihong Zhang, Fanqing Meng, Yan Wang, Yongchang Chen, Yong Xu, Luo Gu

**Affiliations:** ^1^ Cancer Center, Nanjing Medical University, Nanjing, Jiangsu 210029, China; ^2^ Department of Biochemistry and Molecular Biology, Nanjing Medical University, Nanjing, Jiangsu 210029, China; ^3^ Department of Physiology, Nanjing Medical University, Nanjing, Jiangsu 210029, China; ^4^ Department of Biotechnology, Nanjing Medical University, Nanjing, Jiangsu 210029, China; ^5^ Department of Pathophysiology, the First Affiliated Hospital of Nanjing Medical University, Nanjing, Jiangsu 210029, China; ^6^ Department of Pathophysiology, the Affiliated Drum Tower Hospital of Nanjing University Medical School, Nanjing, Jiangsu 210008, China; ^7^ Department of Pathophysiology, the Second Affiliated Hospital of Nanjing Medical University, Nanjing, Jiangsu 210011, China; ^8^ Department of Physiology, School of Medical Science and Laboratory Medicine, Jiangsu University, Zhenjiang, Jiangsu 212013, China; ^9^ Department of Pathophysiology, Nanjing Medical University, Nanjing, Jiangsu 210029, China

**Keywords:** EGF, Arf6, ERK, Wnt5a, EMT

## Abstract

Wnt5a, a ligand for activating the non-canonical Wnt signaling pathway, is commonly associated with Epithelial-to-mesenchymal transition (EMT) in cancer cell metastasis. Here, we show that downregulation of Wnt5a mRNA and protein by EGF is necessary for EGF-induced EMT in gastric cancer SGC-7901 cells. To further explore the mechanisms, we investigated the effect of EGF signaling on Wnt5a expression. EGF increased Arf6 and ERK activity, while blockade of Arf6 activation repressed ERK activity, up-regulated Wnt5a expression and repressed EMT in response to EGF. We also demonstrate that EGF inactivated *Wnt5a* transcription by direct recruitment of ERK to the *Wnt5a* promoter. On the other hand, inhibition of ERK phosphorylation resulted in decreased movement of ERK from the cytoplasm to the nucleus, following rescued Wnt5a mRNA and protein expression and favored an epithelial phenotype of SGC-7901 cells. In addition, we notice that kinase-dead, nuclear-localised ERK has inhibitory effect on Wnt5a transcription. Analysis of gastric cancer specimens revealed an inverse correlation between P-ERK and Wnt5a protein levels and an association between Wnt5a expression and better prognosis. These findings indicate that Wnt5a is a potential suppressor of EMT and identify a novel Arf6/ERK signaling pathway for EGF-regulated Wnt5a expression at transcriptional level of gastric cancer cells.

## INTRODUCTION

Epithelial-mesenchymal transition (EMT) is an essential phenotypic conversion that has been implicated in the initiation of metastasis for gastric cancer progression [1]. When gastric cancer cells receive signals from their microenvironment, such as EGF, TGF-β and hypoxia, cells may lose cell-cell junction, and gain migratory and invasive properties, providing them a distinct advantage in tumor progression and metastasis [2–4]. Despite recent progress, the molecular mechanisms underlying EMT in gastric cancer are not well understood.

Wnt signaling has historically been divided into two categories, namely, the canonical and the non-canonical signaling pathways. Wnt5a belongs to the non-transforming Wnt family that activates the non-canonical Wnt signaling. It is well known that Wnt5a has ample opportunities to influence diverse cell signaling, resulting in functional promiscuity on tumor initiation and progression. For example, in malignant melanoma and pancreatic cancer, Wnt5a promotes tumor progression [5, 6]. However, there are also reports that Wnt5a functions as a tumor suppressor in breast cancer, thyroid cancer, and colon cancer [7–9]. Studies on non-tumorigenic epithelial cells have led to the suggestion that Wnt5a promotes β-catenin/E-cadherin complex formation and that Wnt5a increases intercellular adhesive ability [10]. Multiple studies have highlighted a link between canonical Wnt signaling and EMT, particularly in gastric cancer cells [11, 12]. In addition, it has been revealed that canonical Wnt signaling may be antagonized by Wnt5a in some types of cancer cells [13–15]. Whether Wnt5a can influence the progression of gastric cancer cell by promoting EMT, however, is largely unknown.

*Wnt5a* is known as a highly regulated gene, and multiple transcription factors including NF-κB, GLI, FOX, and SMAD are allowed to bind within the promoter region and play important roles in either promoting or repressing *Wnt5a* transcription under various cellular conditions [16]. After post-translational palmitoylation and glycosylation, Wnt5a is secreted outside the cell and binds to its receptor to exert its biological effects [17]. Normally, it can signal via activation of the Wnt/Ca^2+^ pathway or the Wnt/planar cell polarity pathway to regulate oncogenesis and developmental processes [18]. EGF has been shown to be a potent pro-migratory factor for a variety of cultured gastric cancer cells, and EGFR is highly expressed in gastric cancer [19]. Although Wnt5a transcription can by modulated by multiple mechanisms, such as Hedgehog and TGF-β signaling cascades [16], it remains unclear whether and if so, how EGF can regulate Wnt5a in gastric cancer cells.

Recent studies including the results from our laboratory showed that Arf6 activation could be induced by EGF and act as a mediator of cell migration and invasion in various types of cancer cells [20–23]. Interestingly, an interaction between Arf6 and canonical Wnt signaling has also been suggested to play a role in regulating adhesion junctions in epithelia [24]. In the present study, we investigated the precise role of Arf6 as a mechanistic connection between EGF and Wnt5a expression. We here provide evidence that Wnt5a is a downstream mediator of EGF signaling in gastric cancer cells suggesting a primary effect of Wnt5a on reducing gastric cancer cell EMT. More importantly, we demonstrate that EGF induced activation of Arf6 and its downstream effector ERK, which decreased Wnt5a expression by directly binding to the *Wnt5a* promoter to repress *Wnt5a* transcription. Results obtained in this study clearly establish a new relationship between EGF signaling and Wnt5a transcription in the context of EMT regulation, which could be essential in promoting EMT during invasion and metastasis.

## RESULTS

### EGF induces EMT in gastric cancer SGC-7901 cells

**Figure 1:**
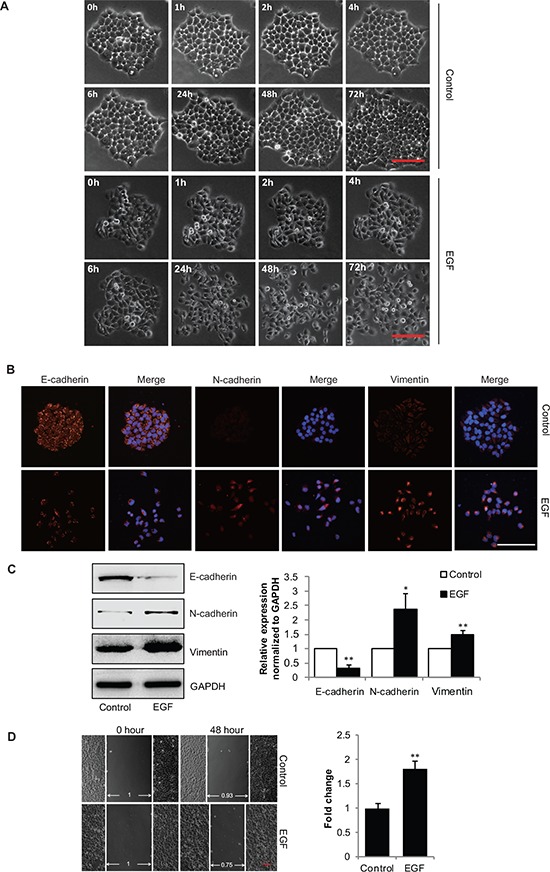
EGF induces EMT in gastric cancer SGC-7901 cells **(A)** SGC-7901 cells were incubated in the absence or presence of EGF (20 ng/mL), cell images were captured by phase-contrast microscopy for indicated times. Scale bar, 100 μm. **(B–D)** The extracts of SGC-7901 cells incubated with EGF (20 ng/mL) for 48 h, (B) representative microscopy images of SGC-7901 cells stained immunofluorescence for E-cadherin, N-cadherin and Vimentin, scale bar, 100 μm, and (C) the total cellular proteins were extracted and analyzed for expressions of E-cadherin, N-cadherin and Vimentin by immunoblotting assays. *P < 0.05, **P < 0.01 in the cultures with EGF relative to the cultures without EGF. (D) The SGC-7901 cells were scraped by a pipette tip and incubated with or without EGF for additional 48 h, a representative of wound healing assay was presented, and the quantification of cell migration rate was performed. **P < 0.01 in the cultures with EGF relative to the cultures without EGF.

To assess the effect of EGF on EMT of gastric cancer cells, SGC-7901 cells were treated with EGF (20 ng/mL) and harvested at indicated time points and the cellular morphologic changes were observed by phase-contrast microscopy. We found that EGF time-dependently induced mesenchymal-like morphologies in SGC-7901 cells (Figure 1A), and led to significant induction of mesenchymal markers Vimentin and N-cadherin. Meanwhile, expression of E-cadherin, an epithelial marker, was decreased after EGF treatment, as shown by immunostaining (Figure 1B) and Western blotting analyses (Figure 1C & Figure S1A–S1B). Functionally, cell motility was increased in response to EGF (Figure 1D). In addition, Our MTT assays also showed that treatment with 20 ng/mL EGF for up to 72 h did not noticeably increase the proliferation of SGC-7901 cells (data not shown). Together, these data suggest that EGF (20 ng/mL) could induce the SGC-7901 cells to undergo EMT-like phenotypic changes. Accordingly, EGF (20 ng/mL) was used for the remainder of the experiments hereafter to identify the mechanism that accounts for the changes in the EMT of SGC-7901 cells.

### Down regulation of Wnt5a is necessary for EGF-induced EMT

**Figure 2:**
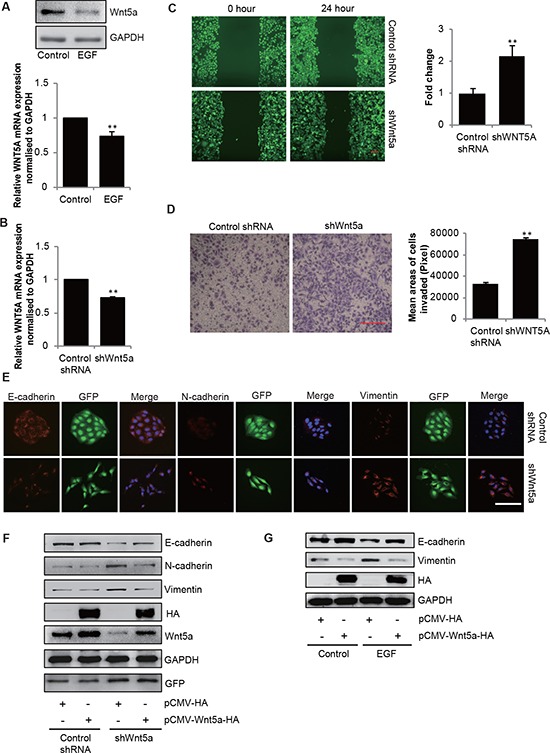
Downregulation of Wnt5a in SGC-7901 cells is necessary for EGF-induced EMT **(A)** Immunoblotting and qPCR analyses of Wnt5a mRNA and protein expressions in SGC-7901 cells that were incubated in the absence or presence of EGF (20 ng/mL) for 48 h. Data are presented as mean ± SD of 3 determinations, **P < 0.01 in the cultures with EGF relative to the cultures without EGF. **(B)** qPCR analyses of Wnt5a mRNA expression in SGC-7901 cells stable transfected with shRNA for Wnt5a. **P < 0.01 in the shWnt5a cells relative to the control group. **(C)** Wound healing assay and **(D)** transwell migration assay of control and Wnt5a knockdown cells. Scale bar, 100 μm. Data are presented as mean ± SD of 3 determinations, **P < 0.01 in the Wnt5a knockdown cells relative to control cells. **(E)** Representative immunofluorescence images of control and Wnt5a knockdown cells stained for E-cadherin, Vimentin and N-cadherin. Scale bar, 100 μm. **(F)** Immunoblotting analyses of E-cadherin, N-cadherin and Vimentin in control and Wnt5a knockdown cells transfected with vector (control) or HA-Wnt5a. **(G)** Cells were transfected with empty vector or HA-tagged Wnt5a, and then incubated with 20 ng/mL EGF for 48 h, the total cellular proteins were extracted and analyzed for expressions of E-cadherin by immunoblotting assays.

We screened the SGC-7901 cells for mRNA expression for all the members of Wnt family and found that Wnt5a was the most abundantly expressed (Figure S2A). In addition, Wnt5a was one of the few members whose mRNA levels were decreased after EGF (20 ng/mL) stimulation for 48 h (Figure S2B). Pretreatment with 20 ng/mL EGF down-regulated Wnt5a expression at both mRNA and protein levels in SGC-7901 cells (Figure 2A). The finding that EGF could block Wnt5a expression prompted us to determine whether Wnt5a expression was required for EGF-induced EMT. Depletion of Wnt5a by shRNA (Figure 2B) induced EMT-like morphological features, such as increased cell motility (Figure 2C & 2D), a spindle-shaped appearance and marked increases in Vimentin and N-cadherin expression as well as simultaneous reduction in E-cadherin expression (Figure 2E & 2F). Further, forced expression of ectopic Wnt5a elevated E-cadherin expression and suppressed Vimentin and N-cadherin expression in shWnt5a cells (Figure 2F). Moreover, restoration of Wnt5a expression partially rescued the reduced expression of E-cadherin by EGF (Figure 2G). Taken together, these experiments demonstrate that down-regulation of Wnt5a is necessary for EGF-induced EMT in SGC-7901 cells.

### Arf6 mediates EGF-induced EMT

**Figure 3:**
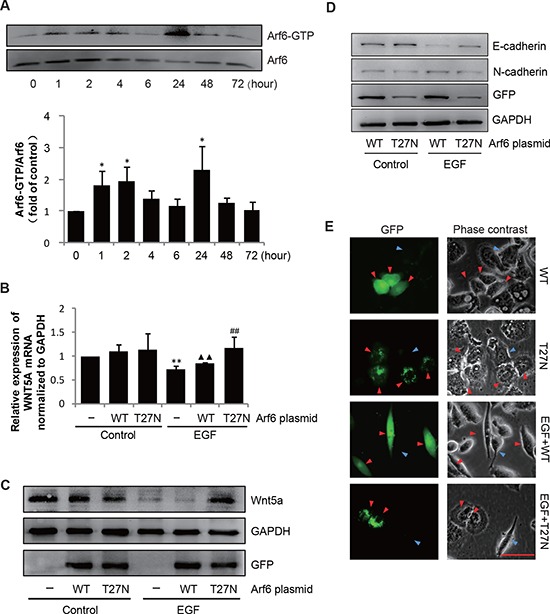
Arf6 mediates the EGF-induced EMT by down-regulating Wnt5a **(A)** SGC-7901 cells were treated with 20 ng/mL EGF for indicated times, and analyzed for Arf6 activity by pulldown and immunoblotting assays. Data are presented as mean ± SD of 3 determinations, *P < 0.05 cultures with EGF relative to the cultures without EGF. **(B–E)** SGC-7901 cells were transfected with Arf6-WT or Arf6-T27N plasmids, then treated with 20 ng/mL EGF for 48 h, (B) mRNA and (C) protein level of Wnt5a, (D) E-cadherin and N-cadherin were detected by qPCR and immunoblotting assay separately. Data are presented as mean ± SD of 3 determinations, **P < 0.01 in cultures with EGF relative to the cultures without EGF. ^▲▲^*P* < 0.01 in the empty vector-expression cells cultured with EGF relative to the cultures without EGF. ^##^*P* < 0.01 in the cells transfected with the Arf6 T27N expression vector treated with EGF relative to the cells transfected with empty vector treated with EGF. (E) The changing shape of cells by EGF was captured by phase-contrast microscopy. Scale bar, 50 μm. Arrows in the panels point to morphology appearance of cells transfected with or without GFP.

Arf6 is well characterized in the EGF pathway, which has been associated with breast cancer invasion [20]. We examined whether Arf6 could also be activated by EGF in SGC-7901 cells. Pulldown assay showed weak but detectable steady state expression of activated Arf6, which was clearly augmentated after 24 h of EGF treatment (Figure 3A). To verify whether Arf6 could impact EGF-mediated EMT, we transfected cells with a dominant negative form of Arf6 (Arf6-T27N), and examined its effect on SGC-7901 cell EMT. As shown in Figure 3B & 3C, following EGF stimulation, the expression of Wnt5a mRNA and protein was decreased significantly by EGF in the control cells transfected with the empty vector, but it was up-regulated in the cells transfected with Arf6-T27N. Meanwhile, Arf6-T27N over-expression partially rescued the reduced expression of E-cadherin and further increased expression of N-cadherin by EGF (Figure 3D), and retained their epithelial feature in spite of EGF stimulation (Figure 3E). Taken together, these experiments demonstrated that Arf6 was required for EGF-induced EMT in SGC-7901 cells likely by repressing Wnt5a expression.

### Phosphorylated ERK mediates EGF/Arf6 signals and regulates Wnt5a mRNA expression via transporting to the nucleus

**Figure 4:**
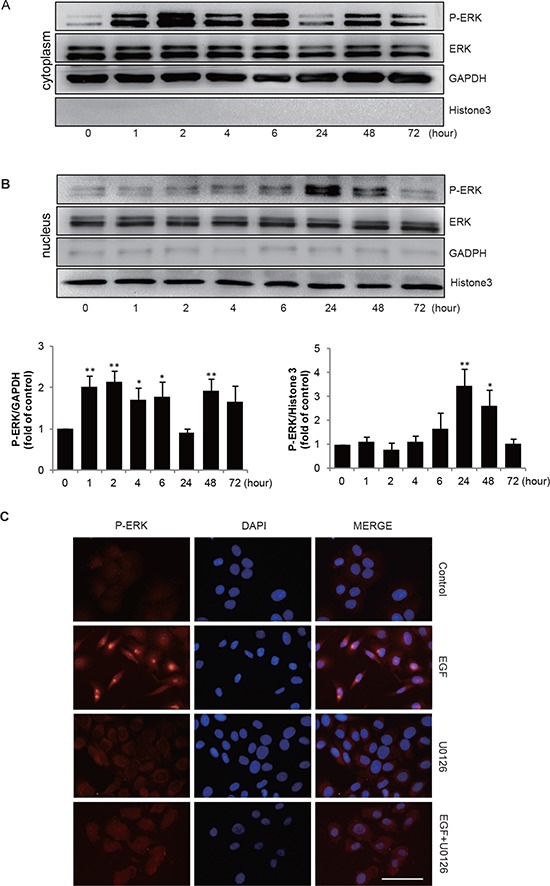
EGF induces P-ERK transportion to nucleaus **(A & B)** SGC-7901 cells were incubated with EGF 20 ng/mL for indicated times, then the extracts of plasma and nuclear section of SGC-7901 cells were subjected to immunoblotting analysis to detect the location and expression of P-ERK separately. GAPDH and Histone 3 are used for detecting cytoplasm and nucleus part. *P < 0.05, **P < 0.01 in the cultures with EGF relative to the cultures without EGF. **(C)** Cells were incubated for 2 h in the absence or presence of 10 μmol/L U0126 prior to EGF treatment (20 ng/mL for 48 h), representative microscopy images of SGC-7901 cells stained immunofluorescence for P-ERK, cell nuclei were labeled with DAPI. Scale bar, 50 μm.

**Figure 5:**
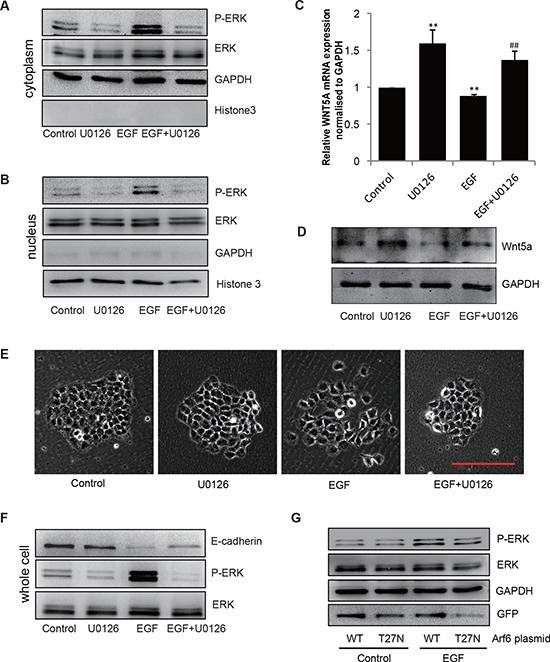
EGF/Arf6 reduces Wnt5a expression and promotes cell EMT through P-ERK **(A–F)** Cells were incubated for 2 h in the absence or presence of 10 μmol/L U0126 prior to EGF treatment (20 ng/mL for 48 h), the extracts of (A) cytoplasm and (B) nucleus were subjected to immunoblotting analysis to detect P-ERK. GAPDH or Histone 3 was as control for cytoplasm or nucleus part. (C) Total mRNA or (D) protein extracts for Wnt5a were analyzed by qPCR and immunoblotting. GAPDH was used as control. **P < 0.01 in the cultures with EGF or U0126 relative to the cultures without EGF. ^##^*P* < 0.01 in the cultures with EGF plus U0126 relative to the cultures with EGF alone. (E) The cell images were captured by phase-contrast microscopy. Scale bar, 100 μm. (F) The extracts of whole cell protein were subjected to immunoblotting analysis to detect E-cadherin. (G) SGC-7901 cells transfected with either an empty vector or an Arf6-T27N expression vector were stimulated with 20 ng/mL EGF for 48 h and ERK activity was examined.

A recent study has reported that ERK acts as a transcriptional repressor for several genes [25]. We hypothesized that P-ERK might also be involved in the down-regulation of Wnt5a in gastric cancer cells in response to EGF. Therefore, we first determined whether EGF could activate ERK phosphorylation in SGC-7901 cells. Immunoblotting assay showed visible cytoplasmic phosphorylation of ERK, which was further increased by EGF stimulation (Figure 4A). Importantly, we were able to detect phosphorylated ERK in the nucleus, which reached peak levels at 24–48 h after EGF treatment as evidenced by immunoblotting (Figure 4B) and immunofluorescence staining assays (Figure 4C). Pre-treatment with MEK kinase inhibitor U0126 inhibited the EGF-induced phosphorylation of ERK both in cytoplasm and nucleus (Figure 4C & Figure 5A–5B).

To probe the involvement of P-ERK in EGF-induced Wnt5a repression in SGC-7901 cells, we treated SGC-7901 cells with U0126 followed by stimulation with EGF. The results showed that U0126 treatment significantly rescued Wnt5a expression at both mRNA and protein levels after EGF stimulation (Figure 5C & 5D). To determine the functional interaction between P-ERK and EMT of SGC-7901 cells, we treated the cells with U0126 and examined EGF-mediated EMT of SGC-7901 cells. Indeed, down-regulation of P-ERK elevated E-cadherin expression and blocked EGF-induced EMT of SGC-7901 cells (Figure 5E & 5F). Similar observations were also made in another gastric cancer cell line (BGC-823, Figure S3A–S3E). We also noticed that Arf6-T27N expression significantly suppressed ERK phosphoyrlation by EGF (Figure 5G), suggesting that ERK might act as a downstream effector of Arf6 in mediating EGF-stimulated gastric cancer cell EMT.

### ERK represses *Wnt5a* transcription in response to EGF stimulation

**Figure 6:**
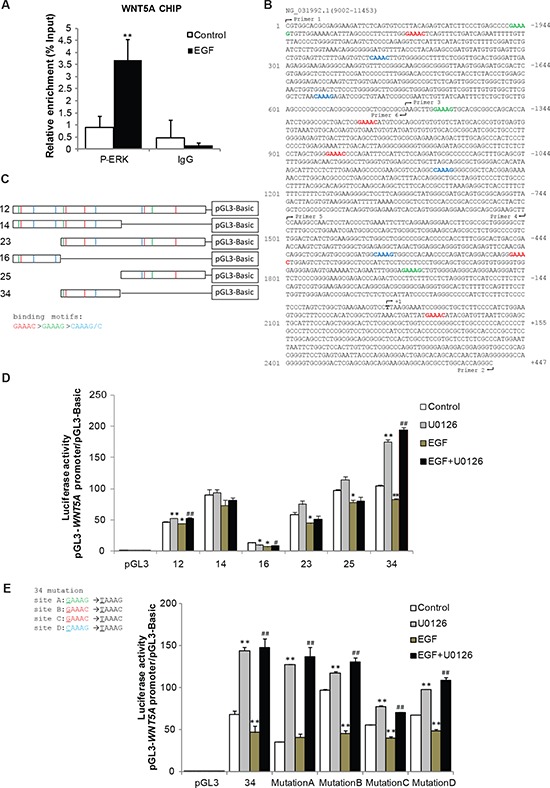
P-ERK binds to *Wnt5a* promoter and represses *Wnt5a* transcription **(A)** ChIP analysis of P-ERK enrichment in the *Wnt5a* promoter region in SGC-7901 cells after EGF (20 ng/mL) treatment for 48 h. **(B & C)** The position of binding motif of P-ERK (G/CAAAG/C) in *Wnt5a* gene promoter is labeled in color, and the primer sets for ChIP analysis are indicated by the arrows in the schematic diagram. **(D)** Luciferase assay measuring the transcriptional activity of different regions on *Wnt5a* promoter (regions on the *Wnt5a* promoter as indicated in C). **(E)** Mutant site of G/CAAAG/C in *Wnt5a* promoter (site A–D) was made and cloned into pGL3 vector. Promoter activity of *Wnt5a* in response to EGF was measured by luciferase assay. Data are presented as mean ± SD of 5 determinations. *P < 0.05, **P < 0.01 in the cultures with EGF or U0126 relative to the cultures without EGF. ^##^*P* < 0.01 in the cultures with EGF plus U0126 relative to the cultures with EGF alone.

**Figure 7:**
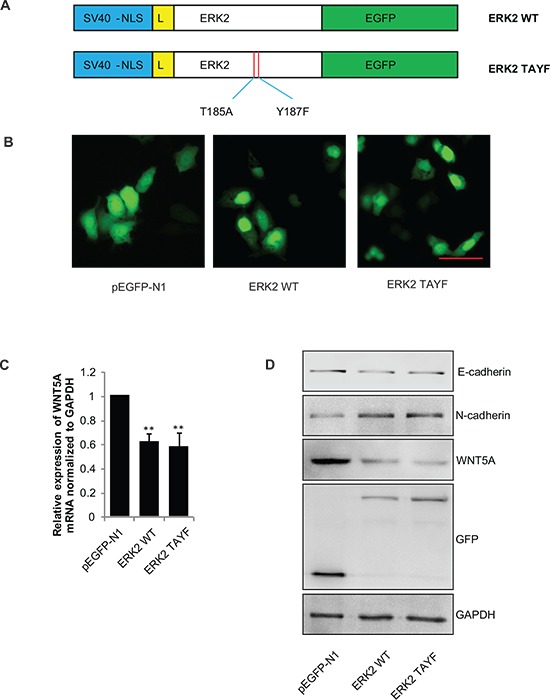
*Wnt5a* transcription and expression requires ERK2 nulear translocation, but not its phosphorylation **(A)** Schematic of the structure of the ERK2 and ERK2-TAYF mutant used for transfection. NLS: nulear localization signal. L:linker sequence. **(B)** Images of the cells transfected with pEGFP-N1, pEGFP-ERK2, and the pEGFP-ERK2-TAYF mutant. Scale bar, 50 μm. **(C)** Total mRNA extracts for Wnt5a were analyzed by qPCR and **(D)** total protein extracts for Wnt5a, E-cadherin and N-cadherin were analyzed by immunoblotting. **P < 0.01 in the cells transfected with the ERK2 and ERK2-TAYF mutant relative to the cells transfected with empty vector.

ERK substrates have a consensus motif G/CAAAG/C that mediates ERK binding [25], and we discovered that there were 12 putative ERK-binding motifs on the *Wnt5a* promoter. Chromatin immunoprecipitation (ChIP) assays demonstrated that P-ERK was indeed specifically interacted with the *Wnt5a* promoter in SGC-7901 cells (Figure 6A). Further, to determine which promoter region of *Wnt5a* plays essential roles in mediating the down-regulation of *Wnt5a* by ERK, we constructed luciferase reporter plasmids bearing either full length of *Wnt5a* promoter or truncated fragments and the constructed plasmids were transfected into SGC-7901 cells (Figure 6B & 6C). We found that pGL3-basic-region 34 displayed markedly decreased luciferase activity by EGF treatment and rescued by U0126 pretreatment (Figure 6D). To further explore which site within region 34 the P-ERK may directly bind to, the nucleotide of each putative ERK motif in region 34 was mutated and then the promoter region were cloned into the parental luciferase reporter construct. We found that mutation of the site A in the *Wnt5a* promoter significantly reduced the transcription activity by EGF stimulation and rescued by U0126 pretreatment (Figure 6E), indicating that binding of P-ERK to this site is critical for the transcriptional activation of *Wnt5a*. Specially, we noticed that an ERK mutant that is kinase-dead but retains the nuclear localization signal (NLS) (Figure 7A) could stay in the nucleus (Figure 7B) and still inhibit *Wnt5a* transcription (Figure 7C) as well as alter the expression of EMT markers (Figure 7D) in gastric cancer SGC-7901 cells. Similar results were obtained from another gastric cancer cell line BGC-823 cell (Figure S4A–S4C). These results suggest that the location of ERK, but not its phosphorylation status, determines its ability to repress *Wnt5a* transcription and promote EMT in SGC-7901 and BGC-823 gastric cancer cells.

### Expression of Wnt5a in gastric cancer correlates with differentiation

**Figure 8:**
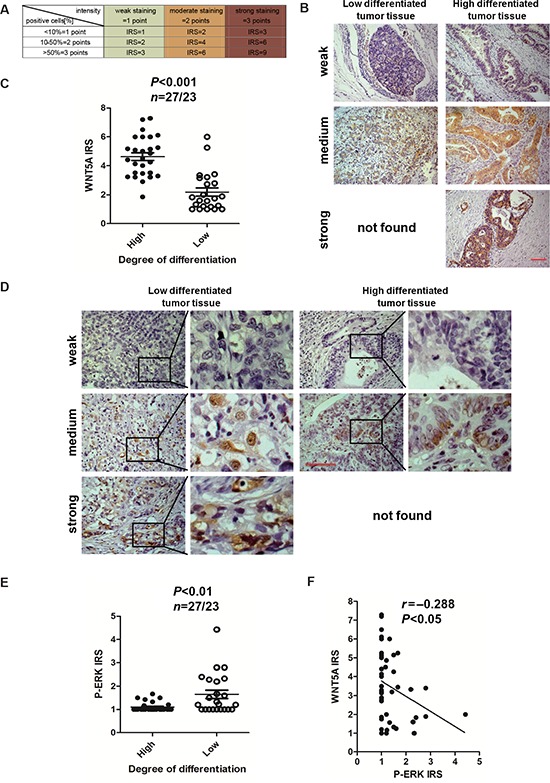
Wnt5a expression is relative with the high degree differentiation of malignant gastric cancer and has a negative correlation with P-ERK **(A)** The IRS was calculated as intensity of the staining reaction multiplied by the percentage of positive cells. Based on the IRS values, Wnt5a and ERK were scored as weak, medium and strong in the following parts. **(B)** High and low degree differentiated malignant gastric cancer tissue sections were stained against Wnt5a. Brown, Wnt5a; Blue, haematoxylin. **(C)** IRS scores of Wnt5a according to tumor histological grade. *P* values and tissue samples are showed above the scatter diagram. **(D)** High and low degree differentiated malignant gastric cancer tissue sections were stained against P-ERK. Brown, P-ERK; Blue, haematoxylin. **(E)** IRS scores of P-ERK according to tumor histological grade. *P* values and tissue samples are showed above the scatter diagram. **(F)** Scatterplot of correlated protein levels between Wnt5a and P-ERK in high and low degree differentiated malignant gastric cancer tissue (*n* = 50). Scale bar, 100 μm.

**Figure 9:**
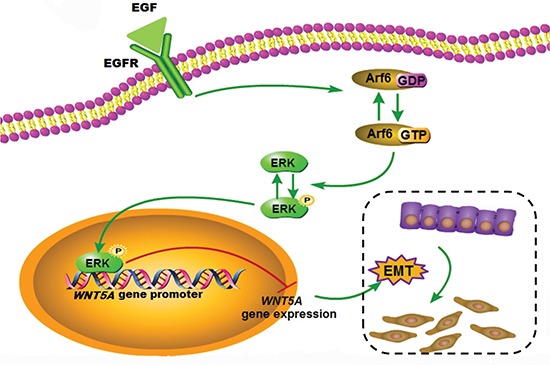
Schematic model for the function of the EGF/Arf6/ERK axis in Wnt5a expression and EMT regulation In summary, we have identified a signaling pathway that is implicated in EGF-induced gastric cancer cell EMT. EGF treatment can lead to the activation of the Arf6/ERK cascade in gastric cancer cells and contribute to P-ERK binding to *Wnt5a* promoter region, thereby suppressing Wnt5a expression at transcriptional level, and low Wnt5a protein expression allows EGF to induce EMT in gastric cancer cells.

To investigate whether our experimental findings could be relevant to the pathogenesis and progression of gastric cancer in humans, we examined Wnt5a and P-ERK expression patterns in gastric cancers with varied degrees of differentiation. The immunoreactive score (IRS) was calculated as the intensity of the staining reaction multiplied by the percentage of positive cells [26, 27] (Figure 8A). Representative results of Wnt5a and P-ERK immunostaining of gastric cancer are shown in Figure 8B & 8D. Based on the analysis of a limited set of 50 gastric cancer cases, we found that the Wnt5a expression level was markedly reduced in poorly differentiated tumor tissues when compared with the well-differentiated tumor tissues, while P-ERK levels were in a reversed pattern (Figure 8C & 8E). In addition, increased nuclear p-ERK staining were observed in poorly differentiated tumor tissues (Figure S5A & S5B). Furthermore, immunostaining of Wnt5a and P-ERK in these 50 primary gastric tumors revealed a negative correlation in expression (*r* = −0.288, *P* < 0.05) (Figure 8F). Finally, increased nuclear p-ERK staining was also inversely correlated with Wnt5a expression (Figure S5A–S5C), suggesting a direct role for ERK in mediating *Wnt5a* transcriptional repression. Overall, our clinical data support our *in vitro* data that Wnt5a may function to oppose gastric cancer progression and ERK may play a role as a Wnt5a repressor (Figure 9).

## DISCUSSION

Both canonical and non-canonical Wnt signalings are associated with the EMT process. Previous studies have shown that aberrant activation canonical Wnt/β-catenin signaling contributes to malignant progression of gastric cancer [28, 29]. Wnt5a is a typical member of Wnt family which activates the non-canonical Wnt signaling pathway. Hence, it is worthwhile to determine the role of Wnt5a on the gastric cancer EMT. To date, only a few factors have been described to regulate Wnt5a expression during gastric cancer progression. In the present study, we provide evidence that inhibition of Wnt5a expression is essential for EGF-induced EMT program in gastric cancer cells. Moreover, down-regulation of Wnt5a is controlled by EGF-mediated activation of Arf6/ERK cascade. Furthermore, we show that nucleus-localized ERK is a transcriptional repressor of Wnt5a. Taken together, we demonstrate for the first time a functional linkage between Arf6-ERK-Wnt5a signaling and induction of the EMT program in gastric cancer cell, which may shed light on new therapeutic targets for gastric cancer.

In contrast to previous findings obtained from human breast cancer, which indicated that Wnt5a mRNA level and protein level are not coordinately regulated in same tissue sample [30, 31], we noticed that gastric cancer SGC-7901 cells are continuously expressing high levels of Wnt5a mRNA and protein, both of which were suppressed by EGF in the present study. In the next set of experiments, we delineated the role of Wnt5a in regulating EMT and cell migration. Our results showed that loss of Wnt5a (shWnt5a) induced EMT and accelerated cell migratory ability. Conversely, gain of Wnt5a expression in shWnt5a cells promoted the acquisition of numerous epithelial characteristics and suppressed migratory behavior. In agreement with these observations, the EGF-mediated increase in EMT-like morphological and expression of several mesenchymal markers were essentially eliminated after Wnt5a over-expression in SGC-7901 cells, indicating that the reversal of the mesenchymal phenotype of SGC-7901 by EGF was specifically due to low Wnt5a expression. Clinically, we have identified that reduced Wnt5a expression are associated with poor differentiation stage of gastric cancer tissue. Together, in the present study, we uncovered an essential role of Wnt5a in regulating EGF-induced gastric cancer cell EMT.

*Wnt5a* gene has been shown to encode two protein isoforms, which exert distinct activities in oncogenesis. The expression of the two Wnt5a isoforms is highly variable among individual cancer cell lines [32]. We noticed that some studies showed that Wnt5a increased migration and aggressiveness of gastric cancer cell lines such as MKN-7 and MKN-74 established in Japan [33–35], so it is possible that the effect of Wnt5a on EMT is gastric cancer cell-type dependent. Given our observation that EGF specifically inhibited Wnt5a expression at the transcriptional level, it will be interesting to elucidate the exact mechanisms by which EGF regulate *Wnt5a* transcription.

Arf6, a member of the ADP-ribosylation factor (Arf) family, has emerged as a critical regulator of membrane trafficking and structural organization at the adhesion junction (AJ) membrane [21, 36]. Since loss of functional AJ is regarded as a hallmark of EMT and cancer cell invasiveness [37], Arf6 may function as a critical determinant of cell-cell AJ disassembly and cancer cell EMT. Our previous work and those of others suggested a link between EGF stimulation and increased Arf6 activity [20, 23]. In fact, it is reported that GEP100, a GEF for Arf6, links EGF/EGFR signaling to Arf6 activation to induce invasive activities of breast cancer cells [22]. Like all GTPases, Arf6 activity is under tight control, which is mediated by guanine nucleotide exchange factors (GEFs) and GTPase-activating proteins (GAPs) that catalyze GTP exchange and hydrolysis, respectively [38]. In the present work, we found that EGF induced Arf6 activation (Arf6-GTP), while blocking Arf6 signaling by dominant negative mutant Arf6-T27N or siRNA for Arf6 greatly abolished EGF-induced cell EMT. These results suggest that EGF might promote EMT in SGC-7901 cells by activating Arf6. Intriguingly, it was recently reported that Wnt5a activates Arf6, leading to the disruption of N-cadherin/β-catenin complexes, and increased melanoma cell invasion [6]. Although the proposal that Wnt5a increases cell migration is compatible with its capacity to increase Arf6 activity in melanoma cells, we have limited knowledge concerning regulation of Wnt5a expression by EGF/Arf6 signaling in gastric cancer cells. Interestingly, we found that Wnt5a mRNA and protein levels were reduced by EGF, which is reversed by inactivation of Arf6 in SGC-7901 cells. Accordingly, we speculated that EGF/Arf6 signals might be able to repress *Wnt5a* transcription directly, thereby allowing EMT in gastric cancer cells. However, by analyzing the genomic sequence of *Wnt5a*, we didn’t identify any potential Arf6-binding sites within the promoter region (data not shown). We therefore speculated that Arf6 might regulate *Wnt5a* transcription indirectly by other factors downstream of Arf6.

Here, we demonstrate that EGF induced a time-dependent increase in ERK activity, which was dependent on Arf6 activation. These results are similar with others’ work using melanoma as well as breast cancer cells [39]. In fact, activation of ERK allows ERK to interact with a nuclear importing protein-importin7, which mediates nuclear ERK translocation [40]. Nuclear localization of ERK may regulate the expression of transcription factors such as c-Fos, SMAD, and p53 [41], so it is worth noting that the subcellular compartmentalization of ERK is an important factor controlling cell fate decisions. Our observations have yielded evidence that that EGF not only enhanced the overall phosphorylation levels of ERK in the cytoplasm but increased the accumulation of p-ERK in the nucleus. Our results also showed that when ERK signaling was blocked, EGF-stimulated Wnt5a mRNA level was increased and cell EMT was dramatically diminished, suggesting that ERK may represent an important mechanism that may directly repress Wnt5a transcription downstream of Arf6.

ERK, an extensively studied protein known for cytoskeletal organization and focal adhesion renewal, has recently been found to be involved in transcriptional regulation [25]. ERK is reported to preferentially bind to chromatin, and the substrates of ERK can directly initiate transcriptional changes [42, 43]. ERK substrates have a consensus motif G/CAAAG/C that mediates ERK binding, and ERK is reported to act as a transcriptional repressor for interferon gamma-induced genes [25]. Through motif analysis, we identified a perfectly matched ERK-binding motif in the *Wnt5a* promoter region. More important, using ChIP and luciferase reporter assays, we were able to verify the importance of ERK within the nucleus, that is, through its direct binding to *Wnt5a* promoter, ERK negatively regulates *Wnt5a* transcriptional expression and serves as a master enforcer of gastric cancer cell EMT. It is believed that the status of ERK phosphorylation may regulate the trans-localization of ERK from the cytoplasm to the nucleus [44]. Interestingly, similar with the observation by Hu et al [25], we noticed that kinase-dead, nuclear-localised ERK2 retained its inhibitory effect on *Wnt5a* transcription in both SGC-7901 and BGC-823 gastric cancer cells. ERK2, but not its isoform ERK1, is suggested to play a more important role in EMT process [45]. Thus, our data suggest *Wnt5a* promoter-binding ability of ERK, possibility that of ERK2, is dependent of its nuclear location, but not its kinase activity.

Although the current study has contributed to the mechanistic understanding the role of Wnt5a in EGF-induced gastric cancer cell EMT, the issue as to how Wnt5a precisely regulates EMT in gastric cancer cells is unlikely to be settled in this paper. Together, these data point to the ability of Arf6/ERK-dependent mechanism that leads to increased EGF-induced EMT of gastric cancer cells by inhibiting *Wnt5a* transcriptional expression. These findings are of potential pathophysiological importance for understanding the integration of migration-related signaling and shed light on new therapeutic targets for gastric cancer.

## MATERIALS AND METHODS

### Cell and plasmids

Human gastric cancer cell line SGC-7901, BGC-823 was obtained from the Cell Biology Institute of Chinese Academy of Sciences (Shanghai, China). SGC-7901 and BGC-823 cells were cultured in Dulbecco's modified Eagle's medium (DMEM, high glucose) (Hyclone, Thermo Scientific, Waltham, MA, USA) supplemented with 10% (v/v) fetal bovine serum (FBS) (Hyclone) and antibiotics (100 U/mL streptomycin and 100 μg/mL penicillin) (Invitrogen, USA) in a humidified incubator at 37°C with 5% CO_2_. Cells were grown on coverslips for fluorescence staining and on plastic dishes for protein extraction. Cells were made quiescent by serum starvation overnight followed by EGF (R&D Systems, Minneapolis, MN, USA) treatment.

The pBIG2r-Wnt5a plasmid and pXS-Arf6 plasmids (WT and T27N) were kindly provided by Dr. P. Michl (Division of Gastroenterology and Endocrinology, Department of Internal Medicine, Philipps-University, Marburg, Germany) and Dr. Julie G. Donaldson (Laboratory of Cell Biology, National Heart, Lung and Blood Institute, National Institutes of Health, Bethesda, Maryland, USA), respectively. Full-length *Wnt5a* cDNA was amplified from pBIG2r-Wnt5a plasmid using the following primer set, sense: 5′-TTGCGGCCGCGCCACCATGAAGAAGTCCATTG GAA-3′, antisence: 5′-CCGGAATTCCTTGCACACAAA CTGGTCCACGATC-3′. In these primers, Not I and EcoR I restriction site sequences have been underlined. The polymerase chain reaction (PCR) products were cloned into the pCMV-C-HA vector (Beyotime, Nantong, China). Full-length *Arf6* cDNA was amplified from pXS-Arf6 using the following primer set, sense: 5′-CCGGAATTCGCCACCATGGGGAAGGTGCTATC- 3′, antisence: 5′-TCCCCGCGGAGATTTGTAGTTAG AGGTTAAC-3′. EcoR I and Sac II restriction site sequences have been underlined. The PCR products were cloned into the pEGFP-N1 vector (CLONTECH Lab, Palo Alto, CA, USA). The ERK2 WT (Genebank NM_002745.4) and TAYF mutation (T185A, Y187F) sequences [46], the SV40 NLS (Nuclear localization sequence): 5′-CCTAAGAAAAAGAGGAAGGTG-3′, and the linker sequence: 5′-GGAGGTGGTGGATC CGGAGGTGGTGGATCCGGAGGTGGTGGATCC-3′ were synthesized and combinated as shown in Figure 7A by GENEWIZ (Beijing, China) and cloned into pEGFP-N1 vector. The cells were seeded in 6-well plates, cultured to 80~90% confluence, and then transiently transfected with those plasmids by using FuGENE HD Transfection Reagent (Promega Corporation, Madison, WI, USA) according to the manufacturer's instructions.

The sequences of small interfering RNA (siRNA) for Arf6 were as follows: #1, 5′-GUGGCAAAUAAUGAGU AAUTT-3′, #2, 5′-GCGACCACUAUGAUAAUAUTT-3′, and #3, 5′-GACGCCAUAAUCCUCAUCUTT-3′; and the sequence of control siRNA was 5′-UUCUCCGAACGUGUCACGUTT-3′ (GenePharma Co., Shanghai, China). Cells were transfected with control siRNA or Arf6 siRNA pool (#1, #2, #3) with Lipofectamine 2000, according to the manufacturer's instruction.

### shRNA knockdown studies

Commercially HIV-based, GFP-labeled and puromycin resistant plasmids Wnt5a shRNA (HSH018531-4-HIVU6) and unrelated control shRNA (CSHCTR001-HIVU6) were obtained from GeneCopoeia (Guangzhou, China). SGC-7901 cells were infected with those lentivirus plasmids by EndoFectin™-Plus (GeneCopoeia) and selected by puromycin (0.4 μg/mL) 48 h after infection.

### Migration assay

SGC-7901 cells were seeded in a 96-well plate. Approximately 48 h later, when cells were 95~100% confluent, cells were incubated overnight in DMEM and wounding was performed by scraping through the cell monolayer with a 10 μL pipette tip. Medium and nonadherent cells were removed, and cells were washed twice with PBS, and new medium with or without EGF was added. Cells were permitted to migrate into the area of clearing for 48 h. For stably transfected cells, the monolayer cells were scratched and allowed to heal for 24 h in DMEM. Wound closure was monitored by visual examination under microscope (Carl Zeiss Meditec, Jena, Germany).

For transwell migration assay, stably transfected SGC-7901 cells in exponential growth were harvested, washed, and suspended in DMEM without FBS. Cells (2 × 10^5^/200 μL) were seeded into polycarbonate membrane inserts (8 μm pore size) in 24-transwell cell culture dishes. Cells were allowed to attach to the membrane for 30 min. The lower chamber was filled with 600 μL DMEM with 10% FBS. Cells were permitted to migrate for 12 h. After the incubation, stationary cells were removed from the upper surface of the membranes. The cells that had migrated to the lower surface were fixed and stained with 0.1% crystal violet. The area of stained cells was counted by pixel in photos taken by Nikon TS100 (Tokyo, Japan).

### RT-qPCR and immunoblotting

Total RNAs were isolated with TRIzol reagent (Invitrogen). Equal amounts of RNA (1 μg) from each sample were used for cDNA synthesis using HiScriptQ RT SuperMix for qPCR (Vazyme, Nanjing, China). qPCR was performed on the ABI StepOne™ Real-Time PCR System (Applied Biosystems, Foster City, CA, USA) using GoTaq qPCR Master Mix assay (Promega) and analyzed using StepOne Software v2.1 (Applied Biosystems). 2−ΔCT method was used to calculate gene expression levels. For sample loading control, GAPDH were tested. Primers used for qPCR amplification are available in Table S1.

Sample protein extraction and concentration determination of whole cells were performed as previously described [47]. Cytoplasmic and nuclear proteins were obtained using the Nuclear and Cytoplasmic Protein Extraction Kit (Beyotime) according to the manufacturer's instructions. Briefly, equal amounts of protein were run on SDS polyacrylamide gels and transferred to nitrocellulose membrane. The resulting blots were blocked with 5% non-fat dry milk and probed with antibodies. The following antibodies were used: GAPDH (KangChen Bio-tech, Shanghai, China), E-cadherin, N-cadherin (BD Transduction Laboratories, Franklin Lakes, NJ, USA), Wnt5a (R&D systems, Minneapolis, MN, USA), Arf6, Vimentin (Abcam, New Terriories, HK, China), ERK, P-ERK, Histone H3, HA and GFP antibodies (Cell Signaling Technology, Danvers, MA, USA). Protein bands were detected by incubating with HRP-conjugated antibodies (Santa Cruz, CA, USA) and visualized with ECL reagent (Millipore, Billerica, MA, USA).

### Pulldown assay

For detection of active Arf6, equal volumes of total cellular protein were incubated with GST-GGA3 for detection of active Arf6 (gifts from James E. Casanova, University of Virginia, VA) beads captured on MagneGST Glutathione Particles (Promega) at 4°C with constant rotation for 30 min. The beads were washed three times with washing buffer (4.2 mmol/L Na_2_HPO_4_, 2 mmol/L KH_2_PO_4_, 140 mmol/L NaCl, and 10 mmol/L KCl, pH7.2). At the end of this period, beads were captured by the magnet in a magnetic stand (Promega) and resuspended in 2 × SDS sample buffer and subjected to immunoblotting analysis by using anti-Arf6 antibody.

### Immunofluorescence and immunohistochemistry assays

Cells used for immunostaining were fixed in ice-cold methanol for 10 min, permeabilized in 0.1% Triton X-100 and blocked in PBS containing 1% BSA for 1 h at room temperature. The cells were incubated with E-cadherin (1:50), N-cadherin (1:50), Vimentin (1:200), P-ERK (1:200) antibodies at 4°C overnight followed by incubation with rhodamine-conjugated anti-rabbit or anti-mouse antibody for 1 h at room temperature within a moist chamber. After wash with PBS, the samples were mounted with DAPI Fluoromount G (Southern Biotech, Birmingham, AL). Images were acquired using an Olympus BX51 microscope coupled with an Olympus DP70 digital camera.

Tumor specimens used were obtained by the First Affiliated Hospital of Nanjing medical University, the Affiliated Drum Tower Hospital of Nanjing University Medical School, Affiliated Zhongda Hospital of Dongnan University and the Second Affiliated Hospital of Nanjing Medical University (Nanjing, China). Fifty primary human gastric tumor samples were used for immunohistological staining in our Wnt5a/P-ERK expression correlation study. The paraffin section were deparaffinized and rehydrated. Peroxidase blocking was done with 3% H_2_O_2_ in methanol for 15 min at 37°C. Antigen retrieval was performed by transferring the sections into Tris-acetate-ethylenediamine tetraacetic acid (EDTA) buffer (pH 8.0) inside a 700-watt microwave oven on full power for 5 min and half power for 10 min. After cooled down to room temperature, the sections were blocked in goat serum for 1 h and applied with Wnt5a (1:600, Novus Biologicals, Littleton, CO, USA) or P-ERK antibody (1:400, Cell Signaling Technology) antibody at 4°C overnight. Then the sections were treated with the secondary antibody (1:1000) for 1 h at 37°C, and then washed in PBS. DAB substrate solution was applied to reveal the color of antibody staining. After counterstained with haematoxylin, the slides were mounted by neutral gum. Reagents for immunohistochemistry were all obtained from ZSGB-BIO (Beijing, China). Wnt5a and P-ERK immunostaining was analyzed by evaluation of the percentage of tumor-stained cells and staining intensity, allowing assessment of an IRS [26, 27]. Blinded scoring was performed independently by authors YJ-Z, H-W and J-C.

### Chromatin-immunoprecipitation

The chromatin immunoprecipitation (ChIP) assay was conducted based on the Millipore-Upstate ChIP protocol with a few modification (Millipore-Upstate, Temecula, CA, USA). Briefly, 1.5 × 10^7^ cells were cross-linked with 1% formaldehyde. The nuclei were collected from the cells and the nuclear chromatin was.sonicated to an average length of about 1 kb. 20% of this chromatin solution was used as input and the remaining was pre-cleared by salmon sperm DNA/Protein A agarose slurry for 30 min at 4°C with rotation. The pre-cleared supernatant was then incubated with P-ERK antibody (1:50, Cell Signaling) or normal rabbit IgG (1:50, Cell Signaling) for 16 h at 4°C. The immune complexes were collected with salmon sperm DNA/Protein A agarose slurry. After wash with gradient stringent buffer, the eluted solution of the complexes was incubated for 16 h with 5 mol/L NaCl at 65°C and then with 0.5 mol/L EDTA, 1 mol/L Tris-HCl and 10mg/mL proteinase K at 45°C to reverse the cross-links. The IP-DNA was extracted using TIANamp Genomic DNA Kit (Tiangen, Beijing, China) and confirmed by qPCR. Primers used for qPCR amplification were as follows: Wnt5a: 5′-GGTTCGGTTTGTGTGGCTTC-3′ (sense), 5′-TGCGACATGTTTCCGAGTCA-3′ (anti-sense); GAPDH: 5′-TACTAGCGGTTTTACGGGCG-3′ (sense), 5′-TCGAACAGGAGGAGCAGAGAGCGA-3′ (anti-sense).

### Luciferase report assay

The Wnt5a promoter [48] was synthesized in pGL3 basic vector by GENEWIZ, Beijing, China. The five short sections of the Wnt5a promoter containing different number of P-ERK binding sites were constructed and also cloned into pGL3 basic vectors (Promega). Primer 1: 5′-GGGGTACCCGTGGCACGCGAGGAAGATT-3′, 2: 5′-GAAGATCTGCCCTGGTGCCAGGCGCTGC-3′; 3: 5′-GGGGTACCAAGCTTGGAAAGTGCACGCG-3′, 4: 5′-GAAGATCTAAGCTTCCGCTGCCGTTCCTCC-3′; 5: 5′-GGGGTACCCCAAGGCCAACTCCTACCCCT-3′, 6: 5′-GAAGATCTCGCGGCGAGCGGGGCGC-3′. The six plasmids were named 12 (full length), 14, 16, 23, 25, 34 (short sections) according to their primers, respectively. In these primers, *Kpn* I and *Bgl* II restriction site sequences have been underlined. pRL-SV40 (Promega) plasmid containing a Renilla luciferase gene was co-transfected as control.

Cells were seeded in 24-well plates 24 h before transfection. The following day, 250 ng of the *Wnt5a* promoter reporter plasmid (full length or short sections) along with 12.5 ng of control plasmid expressing Renilla luciferase was co-transfected using lipofectamine 2000 (Invitrogen). The cells were then treated with EGF (20 ng/mL) or U0126 (10 μmol/L) for 48 h and collected for the luciferase assay using the Dual-Luciferase Assay Kit (Promega) on a GloMax 20/20 luminometer (Promega). Experiments were performed in triplicates and repeated at least three times.

### Statistical analysis

Statistical analysis was performed using the SPSS statistical software program (Version 19.0; SPSS, Chicago, IL, USA). Data were analyzed by Student's *t* test. *P* < 0.05 was considered to be significant (two tailed). Error bars represent standard error of mean (s.e.m.). In immunohistochemistry assay, the difference in expression level of Wnt5a and P-ERK was according to IRS score, Pearson correlation test was used to examine associations between Wnt5a and P-ERK protein expressions.

## SUPPLEMENTARY FIGURES AND TABLE


