# Supplementary material for: EGF-reduced Wnt5a transcription induces epithelial-mesenchymal transition via Arf6-ERK signaling in gastric cancer cells
Source: Oncotarget. 2015 Mar 12;6(9):7244–61. doi: 10.18632/oncotarget.3133 (PMC4466682; doi:10.18632/oncotarget.3133)
Supplement: Supplementary file 1 [file oncotarget-06-7244-s001.pdf]

SUPPLEMENTARY FIGURES AND TABLE

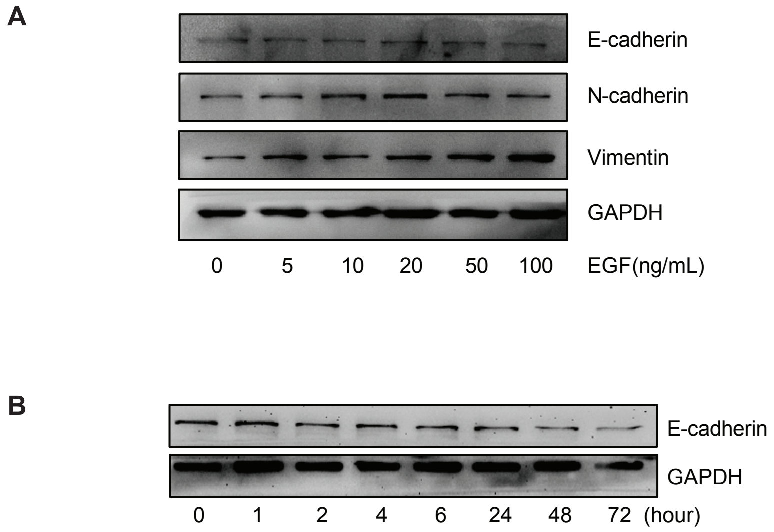

Supplementary Figure S1: Expression of E-cadherin, N-cadherin as well as Vimentin after EGF stimulation in SGC-7901 cells. (A) SGC-7901 cells were cultured in the absence (control) or presence of EGF of indicated concentration for 48 h and E-cadherin, N-cadherin and Vimentin was analyzed by immunoblotting assay. **(B)** SGC-7901 cells were cultured in the absence (control) or presence of EGF of indicated time and E-cadherin expression was analyzed by immunoblotting assay.

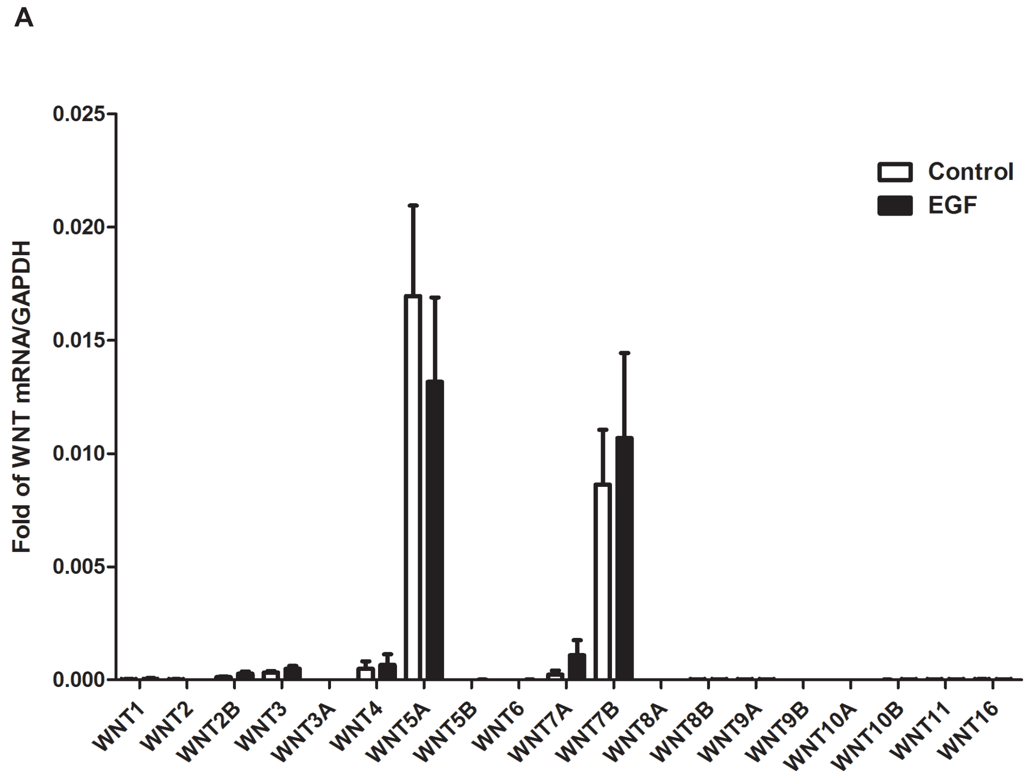

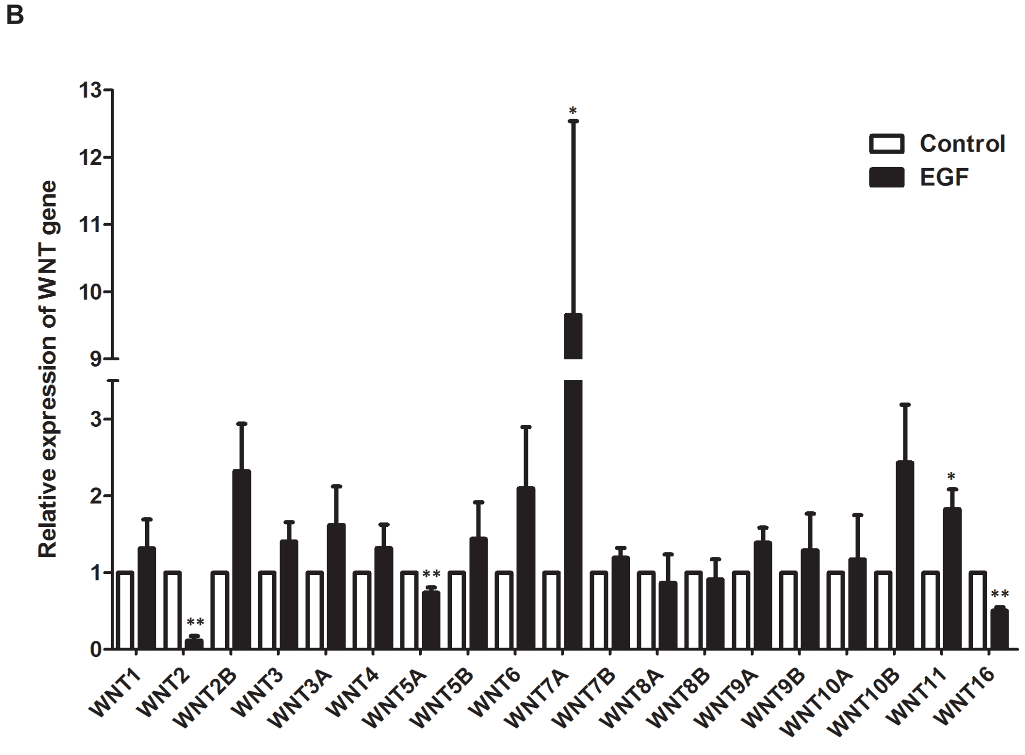

Supplementary Figure S2: Effect of EGF on mRNA expression of Wnt ligands in SGC-7901 cells. SGC-7901 cells were stimulated with EGF (20 ng/mL) for 48 h, and expression of Wnt ligand genes was determined by qPCR. **(A)** Expression of genes is represented corrected for GAPDH **(B)** or relative to control group. Data are presented as mean ± SD of 5 determinations, *$P < 0.05$, **$P < 0.01$ in the cultures with EGF relative to the cultures without EGF.

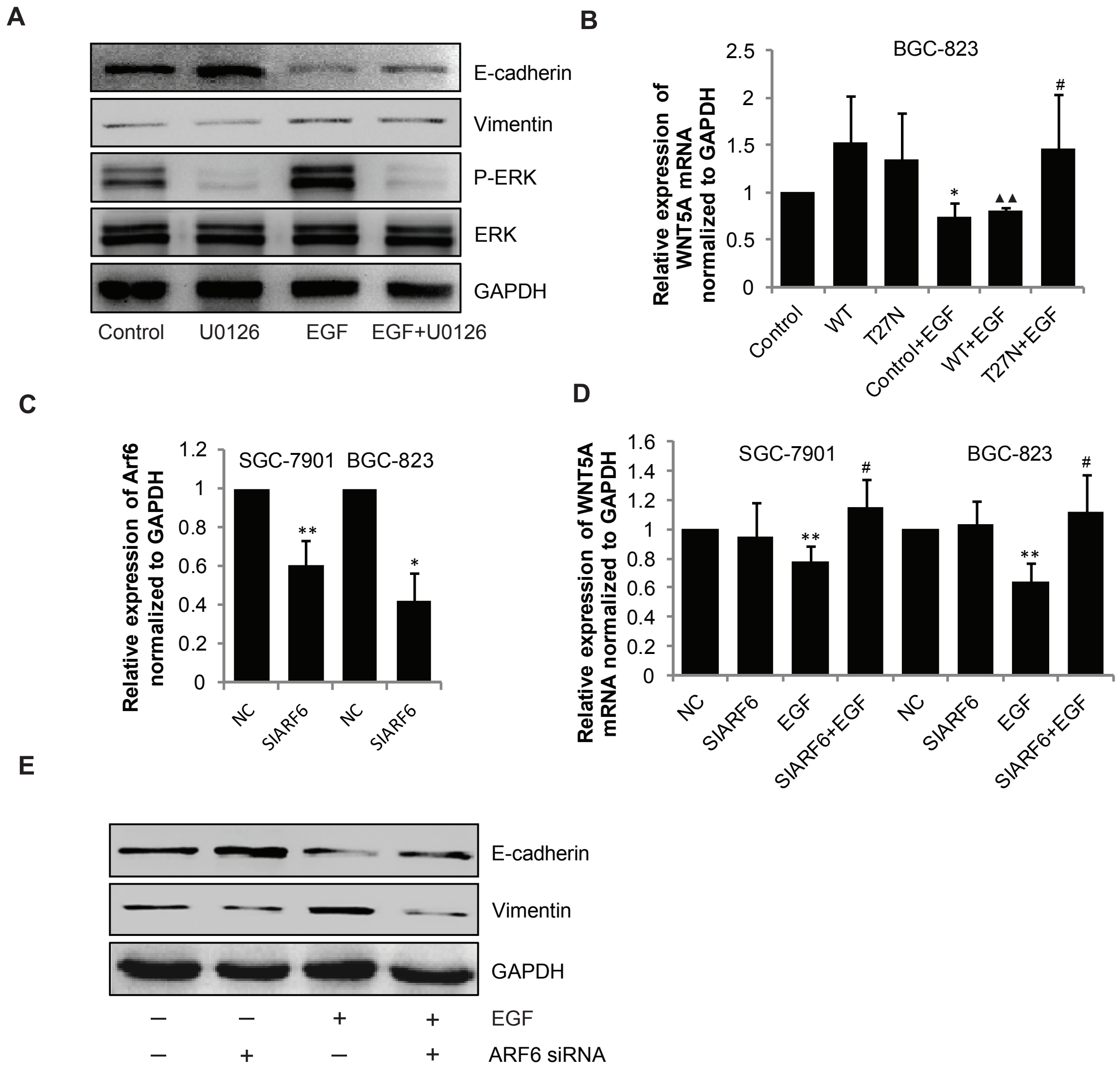

Supplementary Figure S3: Arf6 mediates the EGF-induced Wnt5a low-expression and EMT in BGC-823 cells. (A) Cells were incubated for 2 h in the absence or presence of 10 μmol/L U0126 prior to EGF treatment (20 ng/mL for 48 h), E-cadherin, Vimentin and P-ERK were detected by immunoblotting assay. **(B)** BGC-823 cells were transfected with Arf6-T27N or WT plasmid, then treated with 20 ng/mL EGF for 48 h, mRNA level of Wnt5a were detected by qPCR assay. Data are presented as mean ± SD of 3 determinations, **P* < 0.05 in the cells cultured with EGF relative to the cultures without EGF. ▲▲*P* < 0.01 in the empty vector-expression cells cultured with EGF relative to the empty vector-expression cells without EGF. #*P* < 0.05 in the cells transfected with the Arf6 T27N expression vector treated with EGF relative to the cells transfected with empty vector treated with EGF. **(C–D)** BGC-823 and SGC-7901 cells were transfected with siRNA for Arf6, then treated with 20 ng/mL EGF for 48 h, mRNA level of (C) Arf6 and (D)Wnt5a were detected by qPCR assay. **P* < 0.05, ***P* < 0.01 in the siArf6 cells relative to siRNA control cells. **(E)** protein levels of E-cadherin, Vimentin were detected by immunoblotting separately. Data are presented as mean ± SD of 3 determinations, ***P* < 0.01 in the control siRNA-expression cells cultured with EGF relative to the control siRNA-expression cells without EGF. #*P* < 0.05 in the EGF-treated cells transfected with siArf6 relative to the EGF-treated cells with control siRNA.

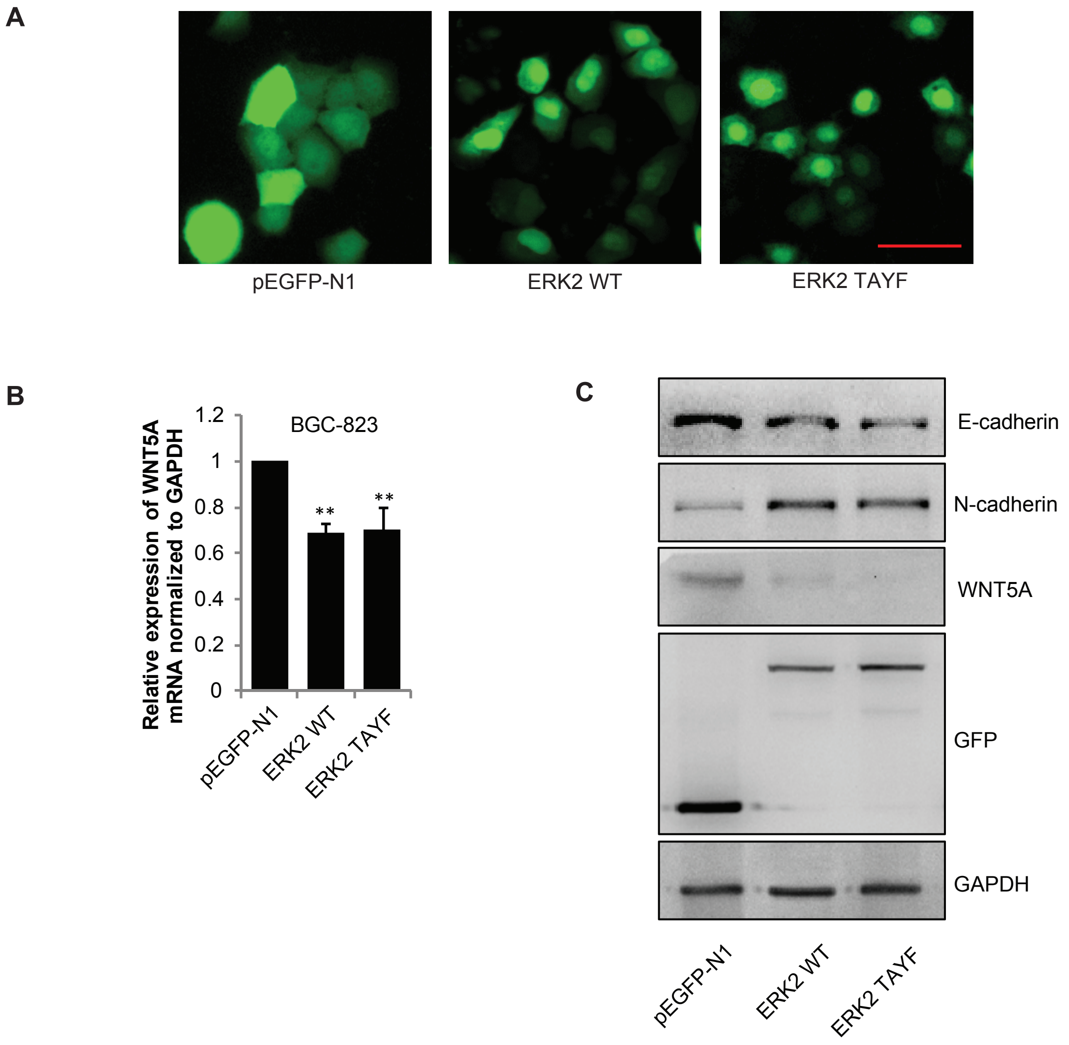

Supplementary Figure S4: *Wnt5a* transcription and expression requires ERK2 nulear translocation, but not its phosphorylation in BGC-823 cells. **(A)** Images of the BGC-823 cells transfected with pEGFP-N1, pEGFP -ERK2, and the pEGFP-ERK2-TAYF mutant. Scale bar, 50 µm. **(B)** Total mRNA extracts for Wnt5a were analyzed by qPCR and **(C)** total protein extracts for Wnt5a, E-cadherin and Vimentin were analyzed by immunoblotting. $^{**}P < 0.01$ in the cells transfected with the ERK2 or ERK2-TAYF mutant relative to the cells transfected with empty vector.

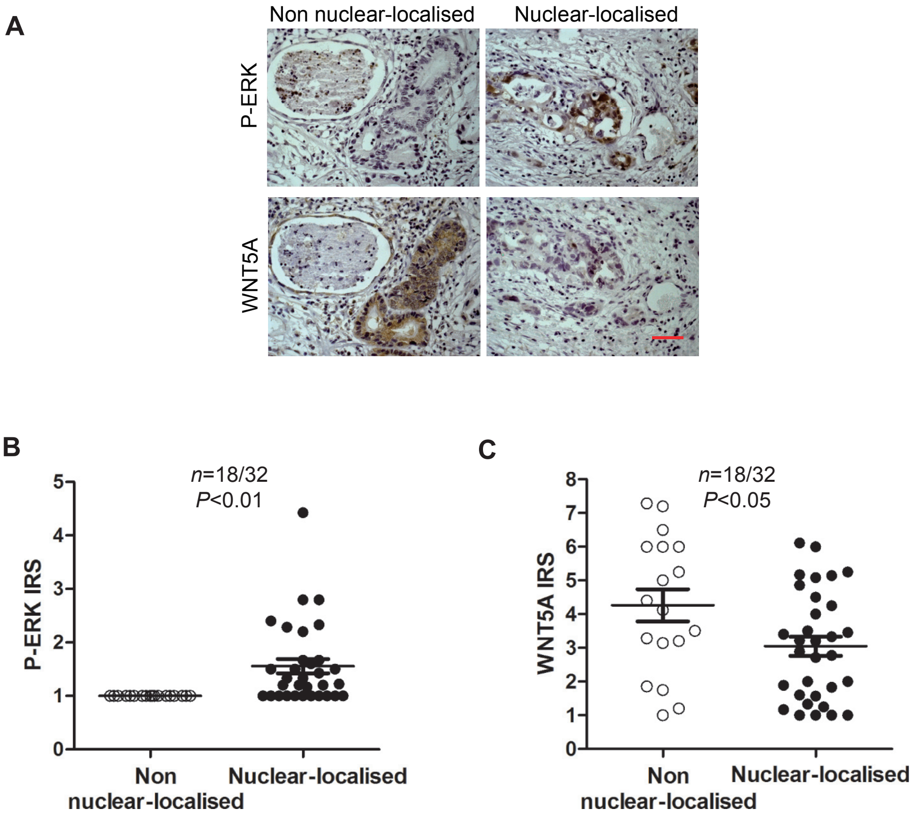

Supplementary Figure S5: Nuclear-localised P-ERK has a negative correlation with Wnt5a expression in gastric cancer. (A) Gastric cancer tissue sections were stained against P-ERK and Wnt5a. Scale bar, 100 µm. **(B)** IRS scores of P-ERK according to its location. **(C)** IRS scores of Wnt5a according to P-ERK location. *P* values and tissue samples are showed above the scatter diagram.

Supplementary Table S1: Primers for q-RCR

WNT1F	GCGACCTCTCTGCCTCTCTTCTT
WNT1R	CCCAACCTCATTTCCACATCATC
WNT2F	GATGCGTGCCATTAGCCAG
WNT2R	AGATTCCCGACTACTTCGGAG
WNT2BF	GGGGCACGAGTGATCTGTG
WNT2BR	GCATGATGTCTGGGTAACGCT
WNT3F	CACCATTTGCGGCTGTGACT
WNT3R	CCTCGTTGTTGTGCTTGTTCAT
WNT3AF	TGCATAGGCTCCTTCCTGTGG
WNT3AR	TGGCTGGTGGGCTGAATTTC
WNT4F	GTACGCCATCTCTTCGGCAG
WNT4R	GCGATGTTGTCAGAGCATCCT
WNT5AF	TGGTGGTCGCTAGGTATGAATAAC
WNT5AR	TCCTGATACAAGTGGCACAGTTTCT
WNT5BF	CGCTTCGCCAAGGAGTTTG
WNT5BR	TGCCATCTTATACACAGCCCT
WNT6F	GGTGCGAGAGTGCCAGTTC
WNT6R	CGTCTCCCGAATGTCCTGTT
WNT7AF	CTGTGGCTGCGACAAAGAGAA
WNT7AR	GCCGTGGCACTTACATTCC
WNT7BF	GAAGCAGGGCTACTACAACCA
WNT7BR	CGGCCTCATTGTTATGCAGGT
WNT8AF	CTACAGAACAGCCACAACACATCC
WNT8AR	ACCTCAGTTTTCCTCTCTTCCACC
WNT8BF	ATCCCACTGGCACTGAGGAGAATA
WNT8BR	GAGTTGGAGGACACAGGTGAAAAGA
WNT9AF	GGCAAGCATCTGAAGCACAAG
WNT9AR	GCAGAAGCTAGGCGAGTCA
WNT9BF	CCACCTGAAGCAGTGTGACCT
WNT9BR	AACTGAAACTGGCACTCAAGCAG
WNT10AF	GGTCAGCACCCAATGACATTC
WNT10AR	TGGATGGCGATCTGGATGC
WNT10BF	CATCCAGGCACGAATGCGA
WNT10BR	CGGTTGTGGGTATCAATGAAGA
WNT11F	GCTGACCTCAAGACCCGATACCT
WNT11R	GACGAGTTCCGAGTCCTTCACAG
WNT16F	AGTATGGCATGTGGTTCAGCA
WNT16R	GCGGCAGTCTACTGACATCAA
GAPDHF	CATCAGCAATGCCTCCTGCAC
GAPDHR	TGAGTCCTTCCACGATACCAAAGTT
ARF6F	CAAAAGGGTATTCTCATGTAGGCT
ARF6R	GTGTTCTTAGATGTGTTTATCAGCC
